# Characterization of the subcellular localization of Epstein-Barr virus encoded proteins in live cells

**DOI:** 10.18632/oncotarget.19549

**Published:** 2017-07-25

**Authors:** Mingsheng Cai, Zongmin Liao, Tao Chen, Ping Wang, Xingmei Zou, Yuanfang Wang, Zuo Xu, Si Jiang, Jinlu Huang, Daixiong Chen, Tao Peng, Gengde Hong, Meili Li

**Affiliations:** ^1^ Guangdong Provincial Key Laboratory of Allergy and Clinical Immunology, Second Affiliated Hospital of Guangzhou Medical University, Guangzhou 510260, Guangdong, China; ^2^ Department of Pathogenic Biology and Immunology, Sino-French Hoffmann Institute, School of Basic Medical Science, Guangzhou Medical University, Guangzhou 511436, Guangdong, China; ^3^ Guangdong Haid Group Co., Ltd., Guangzhou 511400, Guandong, China; ^4^ The Third Clinical School of Guangzhou Medical University, Guangzhou 510150, Guangdong, China

**Keywords:** Epstein-Barr virus, subcellular localization, live cell microscopy

## Abstract

Epstein-Barr virus (EBV) is the pathogenic factor of numerous human tumors, yet certain of its encoded proteins have not been studied. As a first step for functional identification, we presented the construction of a library of expression constructs for most of the EBV encoded proteins and an explicit subcellular localization map of 81 proteins encoded by EBV in mammalian cells. Viral open reading frames were fused with enhanced yellow fluorescent protein (EYFP) tag in eukaryotic expression plasmid then expressed in COS-7 live cells, and protein localizations were observed by fluorescence microscopy. As results, 34.57% (28 proteins) of all proteins showed pan-nuclear or subnuclear localization, 39.51% (32 proteins) exhibitted pan-cytoplasmic or subcytoplasmic localization, and 25.93% (21 proteins) were found in both the nucleus and cytoplasm. Interestingly, most envelope proteins presented pan-cytoplasmic or membranous localization, and most capsid proteins displayed enriched or complete localization in the nucleus, indicating that the subcellular localization of specific proteins are associated with their roles during viral replication. Taken together, the subcellular localization map of EBV proteins in live cells may lay the foundation for further illustrating the functions of EBV-encoded genes in human diseases especially in its relevant tumors.

## INTRODUCTION

Epstein-Barr virus (EBV), a representative member of family gammaherpesvirus (large DNA virus) that can afford interesting model system for viral-host interactions, is related with some severe human health problems such as infectious mononucleosis, various malignancies including nasopharyngeal carcinoma (NPC), gastric carcinoma, diffuse large B-cell lymphoma, NK/T-cell lymphomas, as well as Hodgkin Lymphoma (HL), Burkitt Lymphoma (BL) and post-transplant lymphoproliferative disease (PTLD) [1–3]. The EBV genome is composed of linear double-stranded DNA, about 172 kilobase pairs (kb) in length. Until now, it is reported that EBV encodes approximately 80 to 86 proteins (depending on the strain) [4–7], among which at least 33 are structural components of the virion [8]. However, the absolute number of EBV-encoded proteins is still under investigation because of the examination of different gene transcript products in varied types of infected cells [4, 7, 9–14]. In terms of their localization in the virions, the proteins could be divided into five groups: envelope, tegument, capsid, and unclassified and nonstructural proteins. This complicated network of proteins leads to various consequences on the host cell and guarantees efficient proliferation of the virus.

Although the features of a set of these proteins are already well studied, there is little or nothing report concerning the role of many of the proteins. Therefore, more exhaustive analyses might contribute to figure out the complexity of the EBV pathogenic repertoire. A better perceiving of the individual protein functions is not only conducive to establish the complex replication and potentially approaches for inhibition of propagation, but also helpful for revealing the mechanisms that manipulate vital cellular processes by EBV. Subcellular localization of a protein reflects and is closely correlated with its function, and establishment of the subcellular localization has been demonstrated to be a helpful fashion to estimate the potential functions of a great number of proteins [15], where individual proteins were expressed fused to epitope or to GFP tags [16, 17]. For most proteins, localization results were disclosed to be consistent in different survey regardless of the tag used or expression level. Subcellular localization screening is also a proper starting point to identify the roles of a certain protein of herpesvirus, giving insight into the potential effect of the protein in the course of infection, as well as the cellular processes that may be regulated.

The subcellular localization of part of the EBV-encoded proteins is not known yet. To acquire a more comprehensive knowledge of the functions of EBV proteins, we constructed a mammalian expression library that is composed of most of the open reading frames (ORFs) of EBV. These proteins fused to the C terminus of enhanced yellow fluorescent protein (EYFP) tag that reasonable for protein localization exploration were expressed in COS-7 live cells, and we presented the subcellular localization for all of these EBV-encoded proteins.

## RESULTS

As a substantial step to figure out the detailed function of EBV-encoded proteins, a research has been performed to establish the genome-wide subcellular localization map of EBV-encoded proteins. To investigate the subcellular localization, almost each of the predicted ORFs from EBV, were isolated, and inserted into the expression plasmid, in frame fused with the C terminus of a 27 kDa EYFP tag-encoding sequence, which is demonstrated may but not exert significant influences on protein subcellular localization and favourable for direct fluorescence detection of the respective proteins in live cells. The constructed plasmids were confirmed and transfected into COS-7 cells for expression. COS-7 cells were used as a cell system for these studies because they exhibit a larger cytoplasm than HEK293 cells, which are the more commonly utilized cell types for subcellular localization study. Furthermore, the subcellular localizations of some representative EBV proteins fused with Flag tag were also detected using indirect immunofluorescence (IFA), to make the data more convincing. To get a general overview of the subcellular localization of each protein, Zeiss Axiovert 200M microscope was employed. With this procedure the EBV proteins could be clearly observed in considerable transfected cells. Although not every potential ORF was recovered in the high through-put cloning effort, we produced expression constructs for the majority of EBV, namely 81 EBV ORFs. Among these EBV ORFs, 78 were cloned from B95-8 strain of EBV (174-kb BAC), and the rest ORFs (*LF1*, *LF2* and *LF3*), which could not isolate from 174-kb BAC, were cloned from the BAC DNA of EBV Akata strain of EBV (AK-BAC).

**Figure 1:**
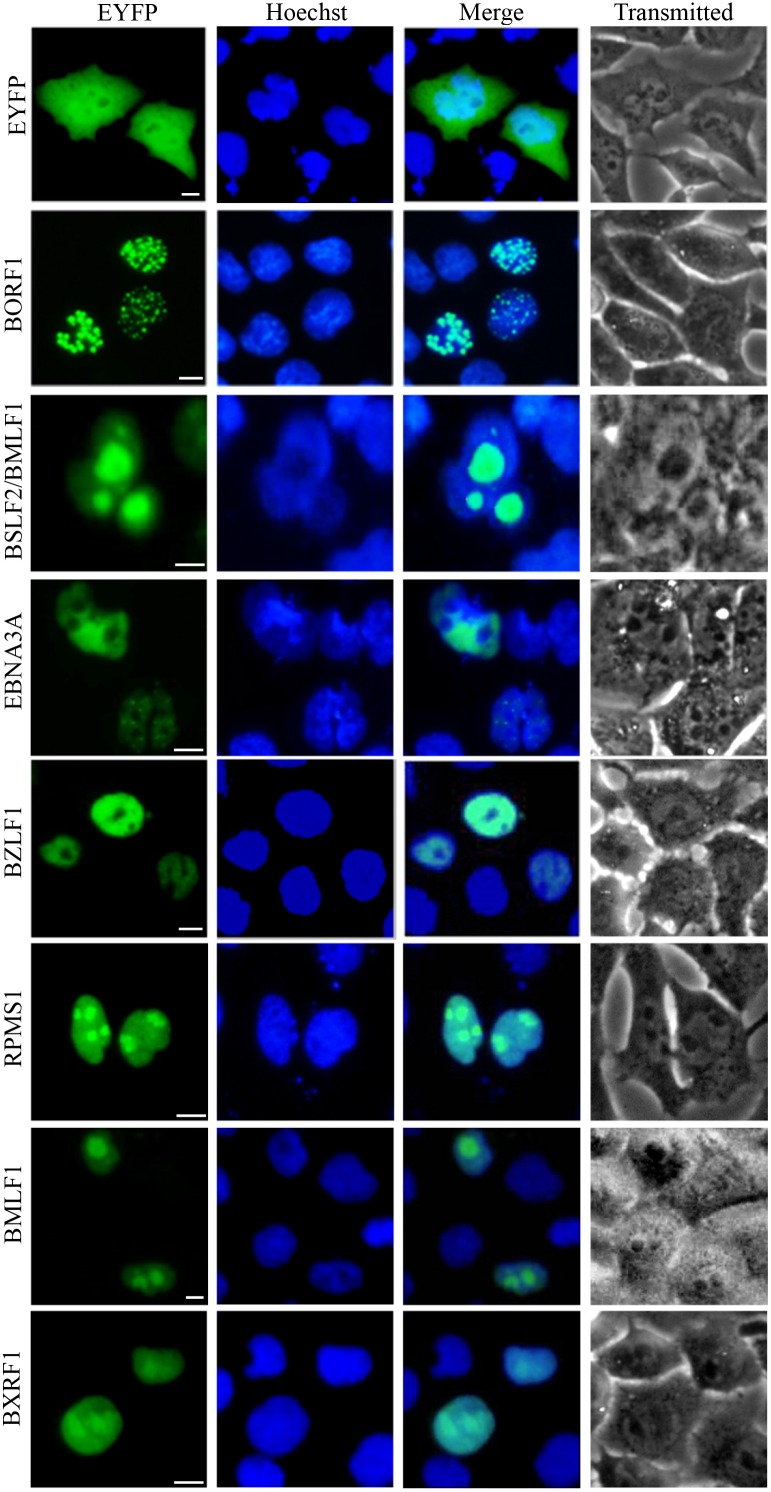

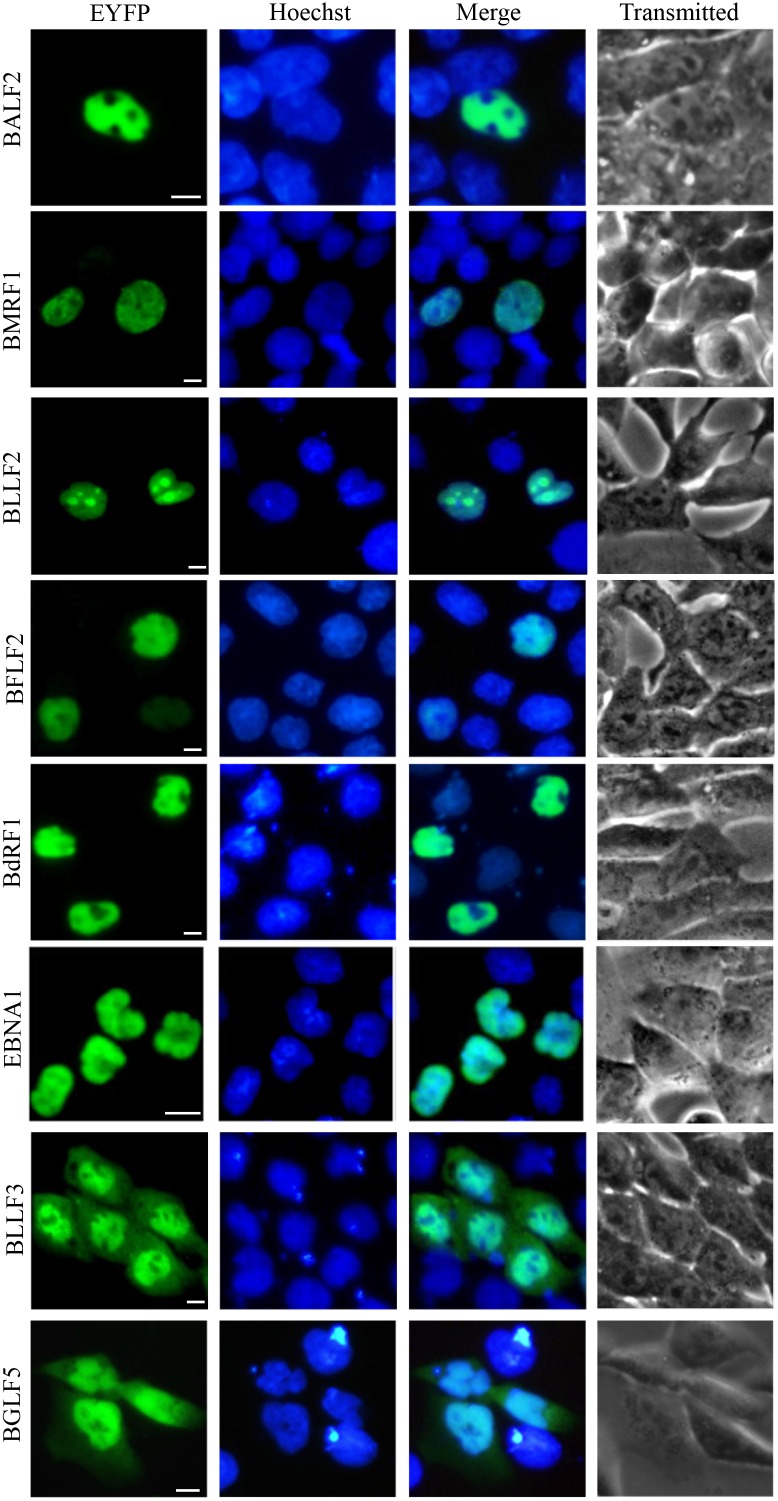

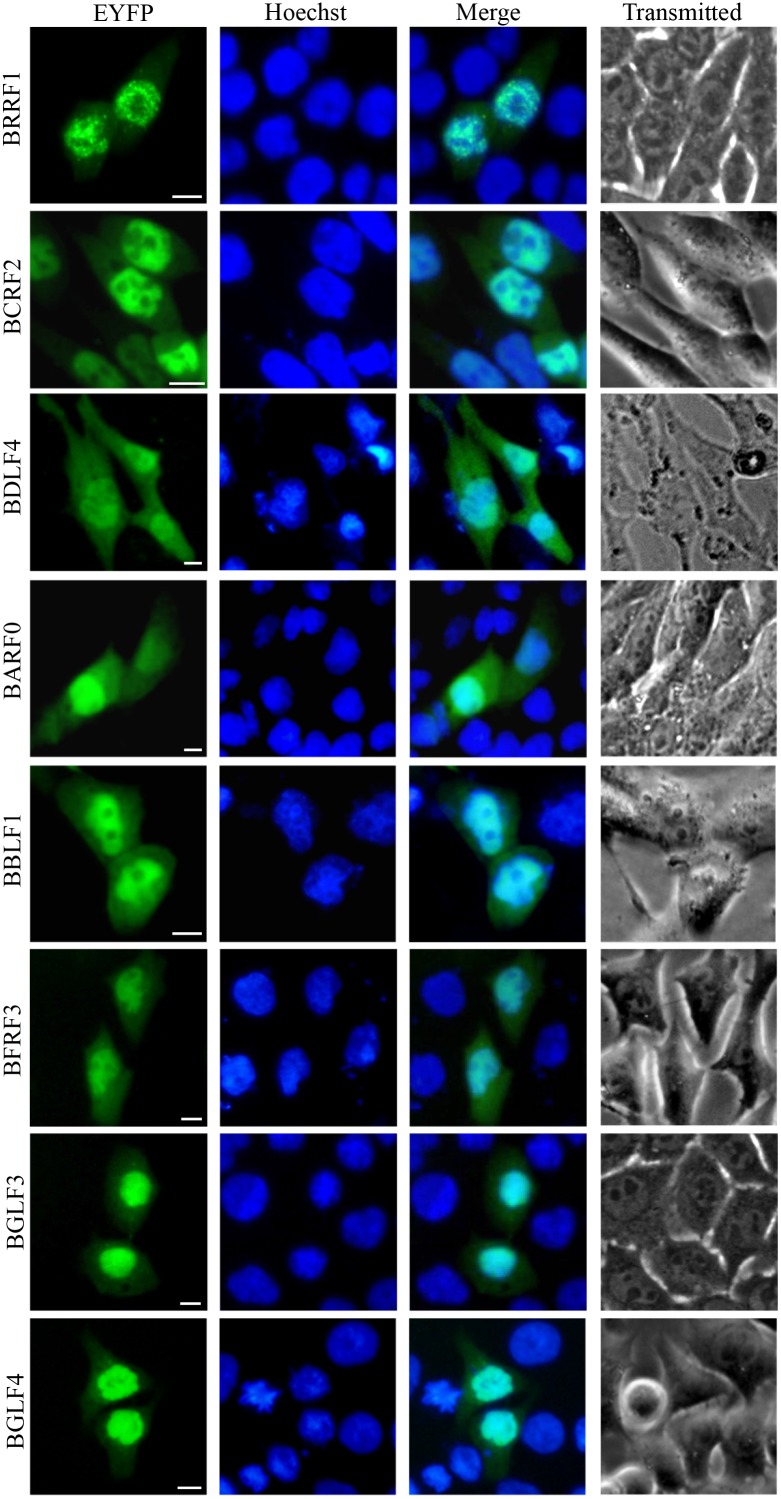

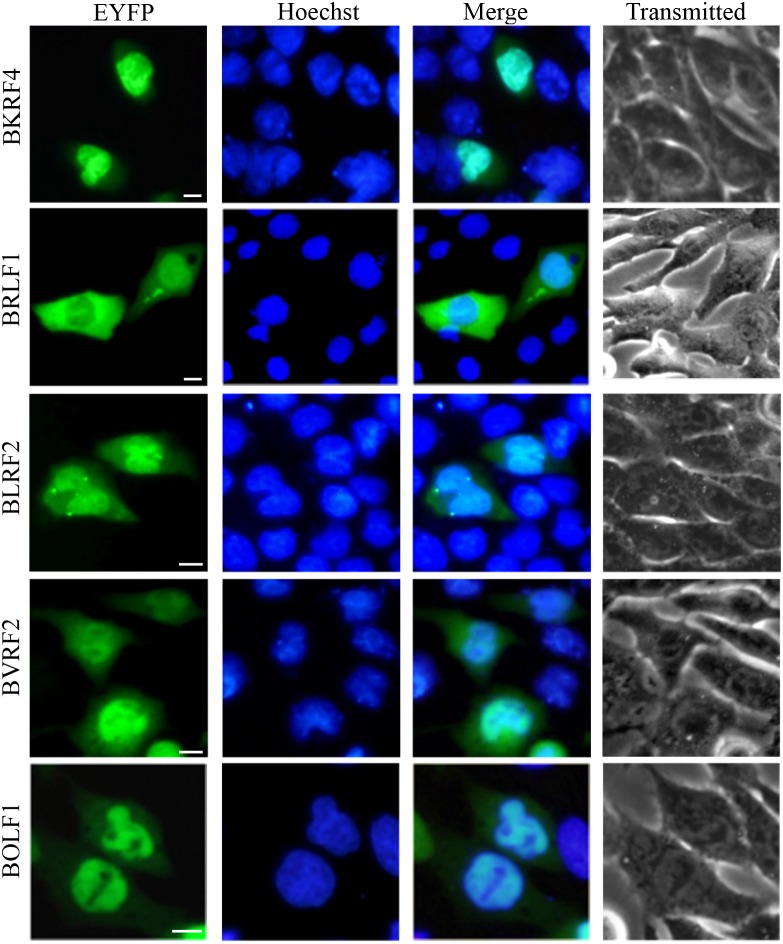
Nuclear localization summary of EBV-encoded proteins 28 EYFP-fused EBV proteins were expressed in COS-7 cells, and cells were subjected to fluorescence microscope analysis in live cells 24 h after transfection. As a negative control, cells were transfected with the vector control (pEYFP-C1). Pictures were obtained using a Zeiss Axiovert 200M microscope. The same magnification was used in all panels. Representative fluorescence images of the vast majority live cells expressing indicated fusion protein were shown. Cells were counterstained with Hoechst to visualize the nuclear DNA. All scale bars indicate 10 μm.

**Figure 2:**
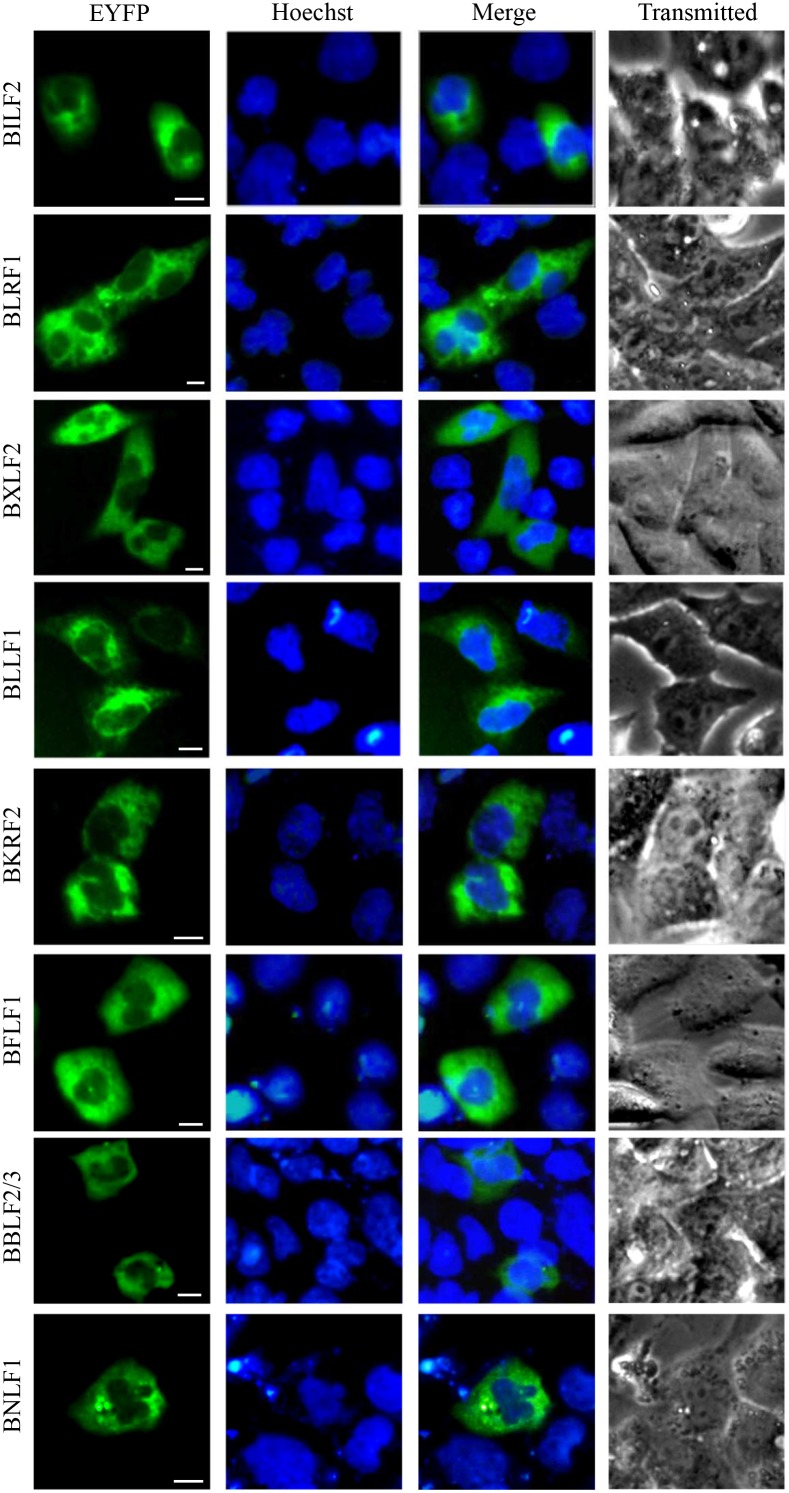

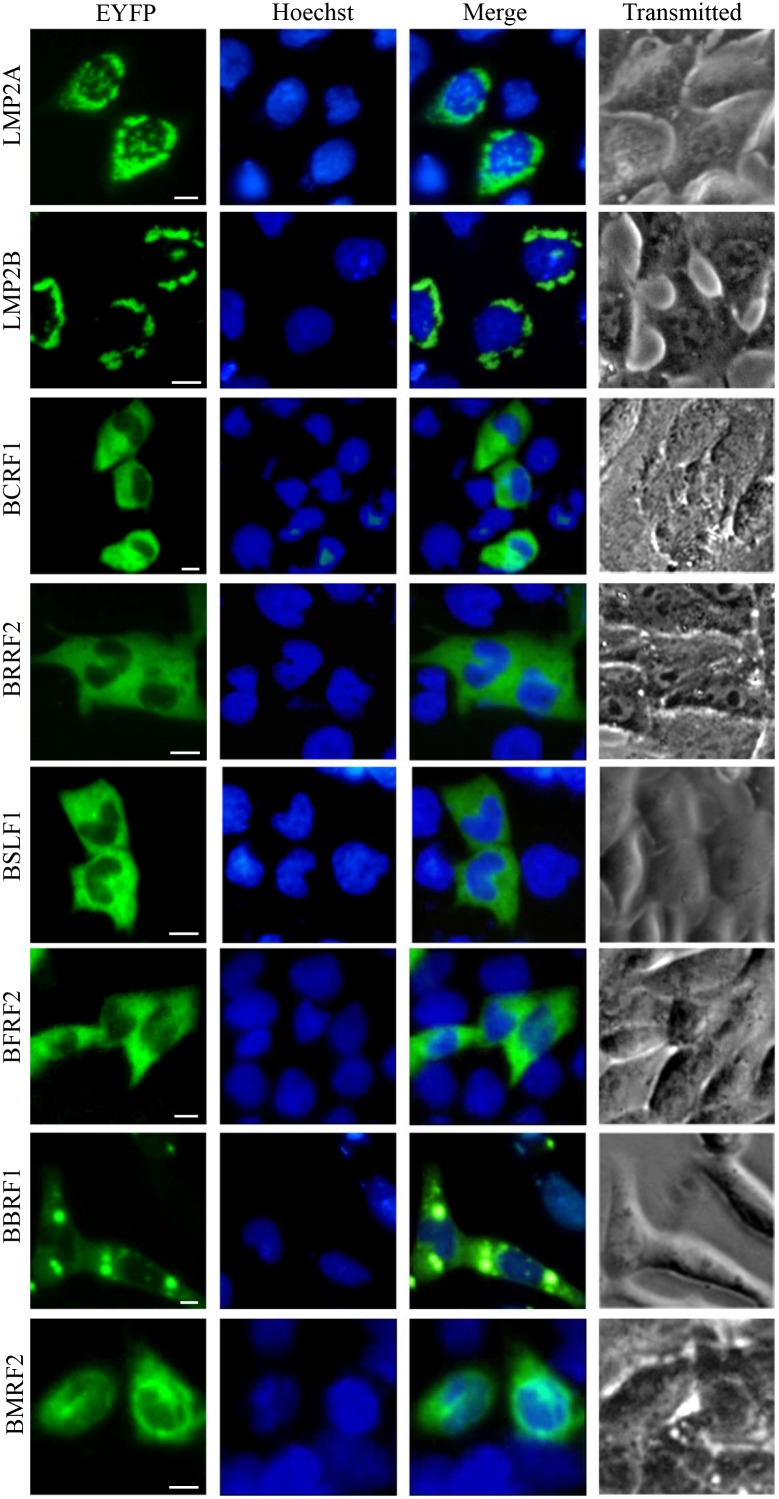

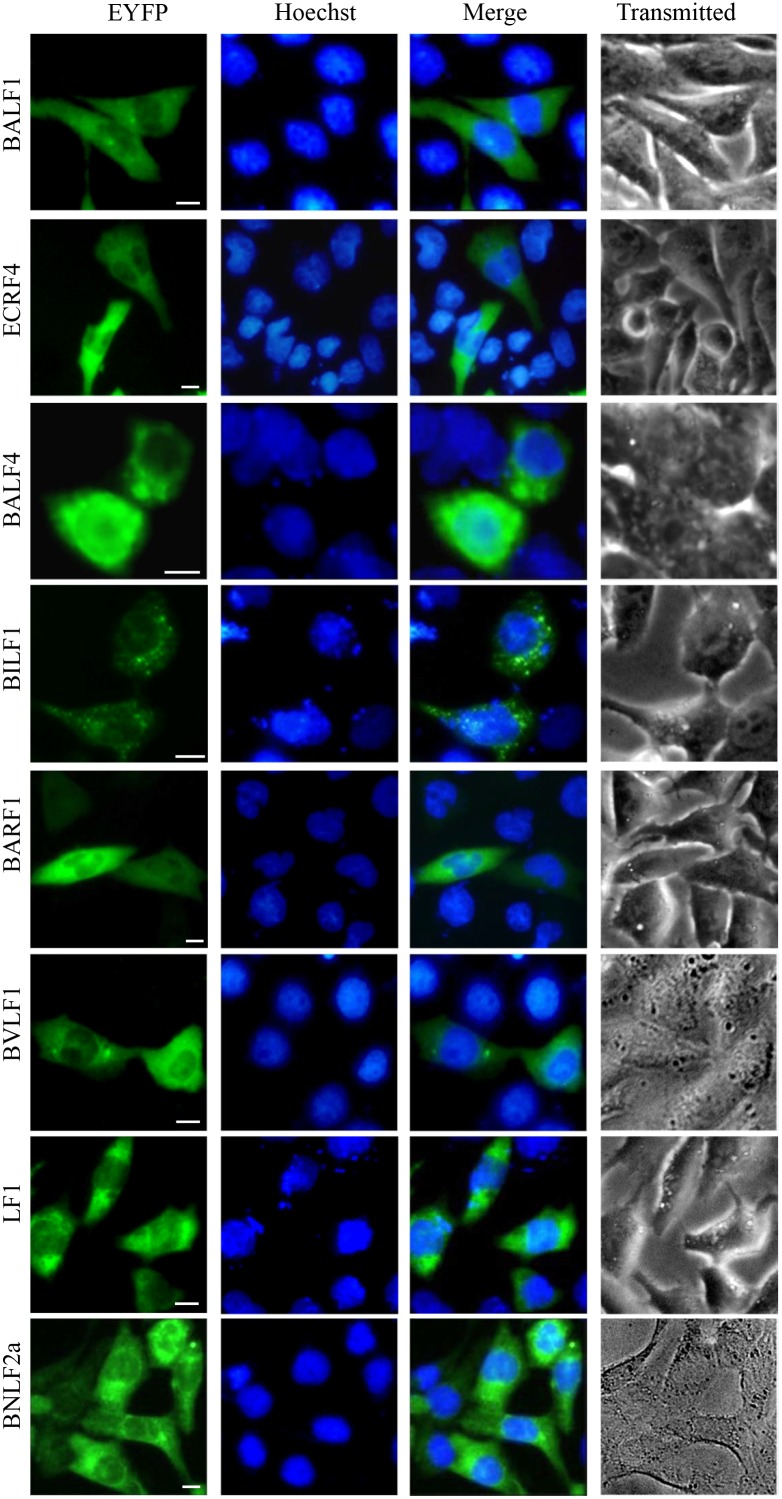

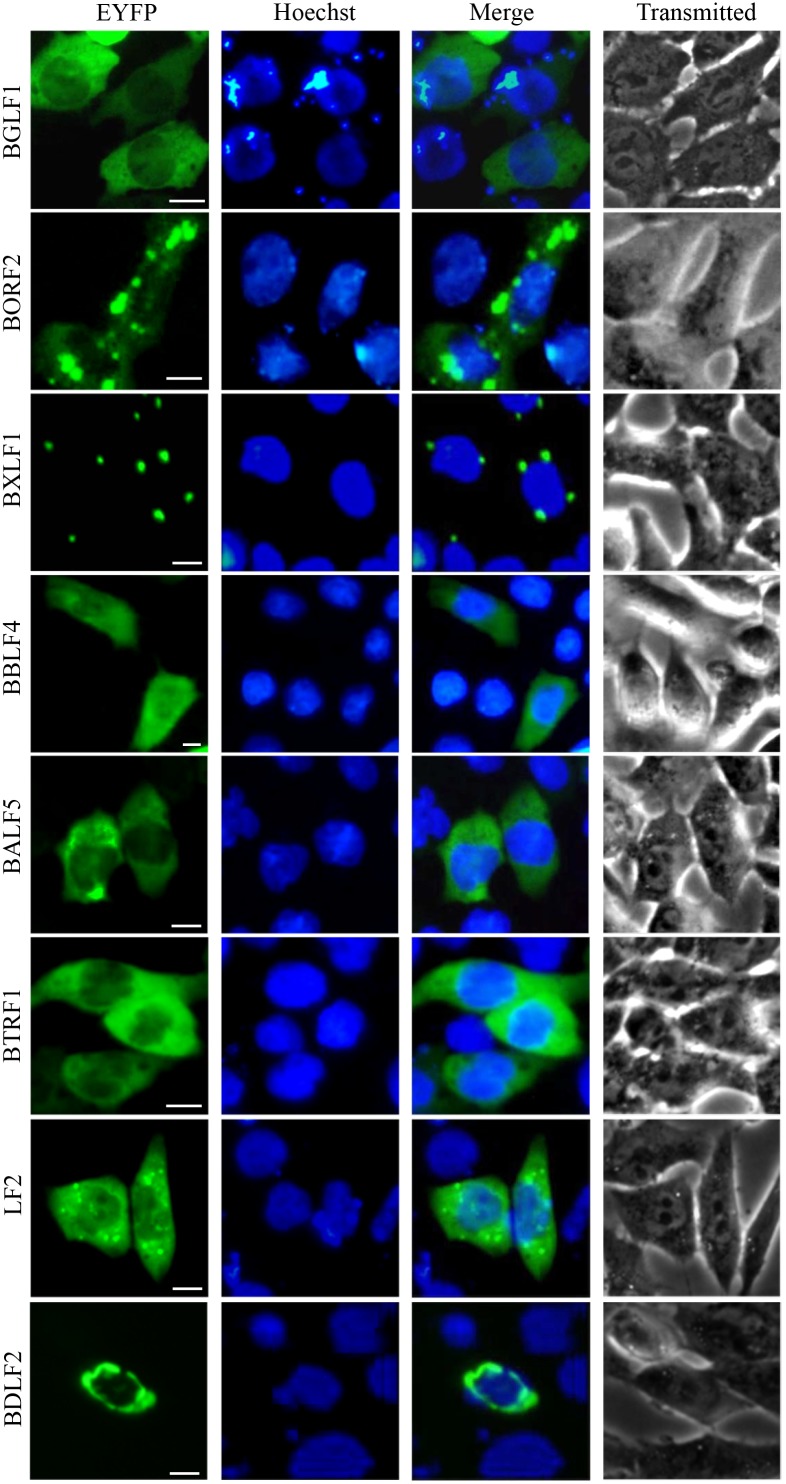
Cytoplasmic localization summary of EBV-encoded proteins 32 EYFP-fused EBV-encoded proteins were expressed in COS-7 cells, and cells were subjected to fluorescence microscope analysis in live cells 24 h after transfection. Pictures were obtained using a Zeiss Axiovert 200M microscope. The same magnification was used in all panels. Representative fluorescence images of the vast majority live cells expressing indicated fusion protein were shown. Cells were counterstained with Hoechst to visualize the nuclear DNA. All scale bars indicate 10 μm.

**Figure 3:**
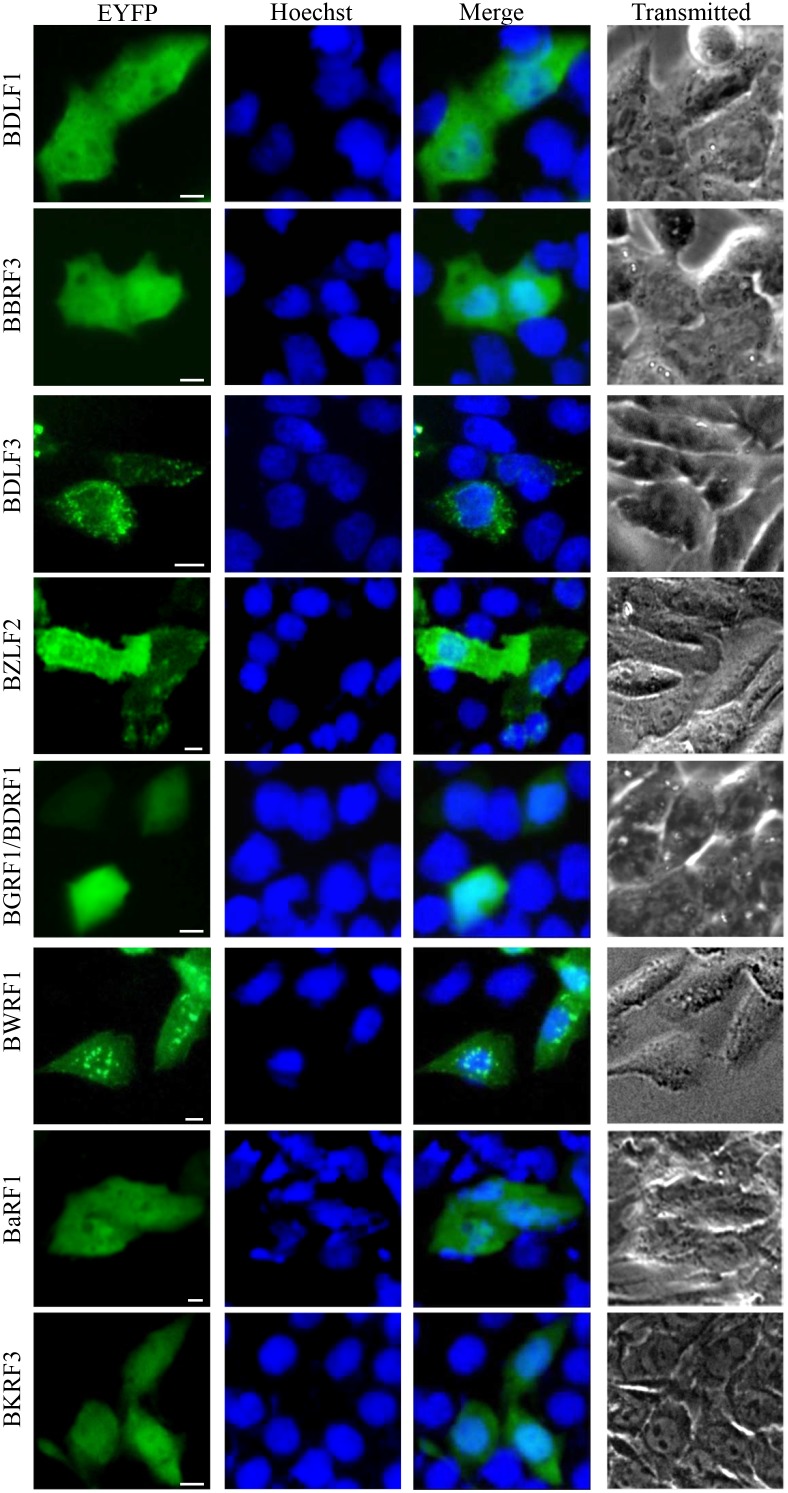

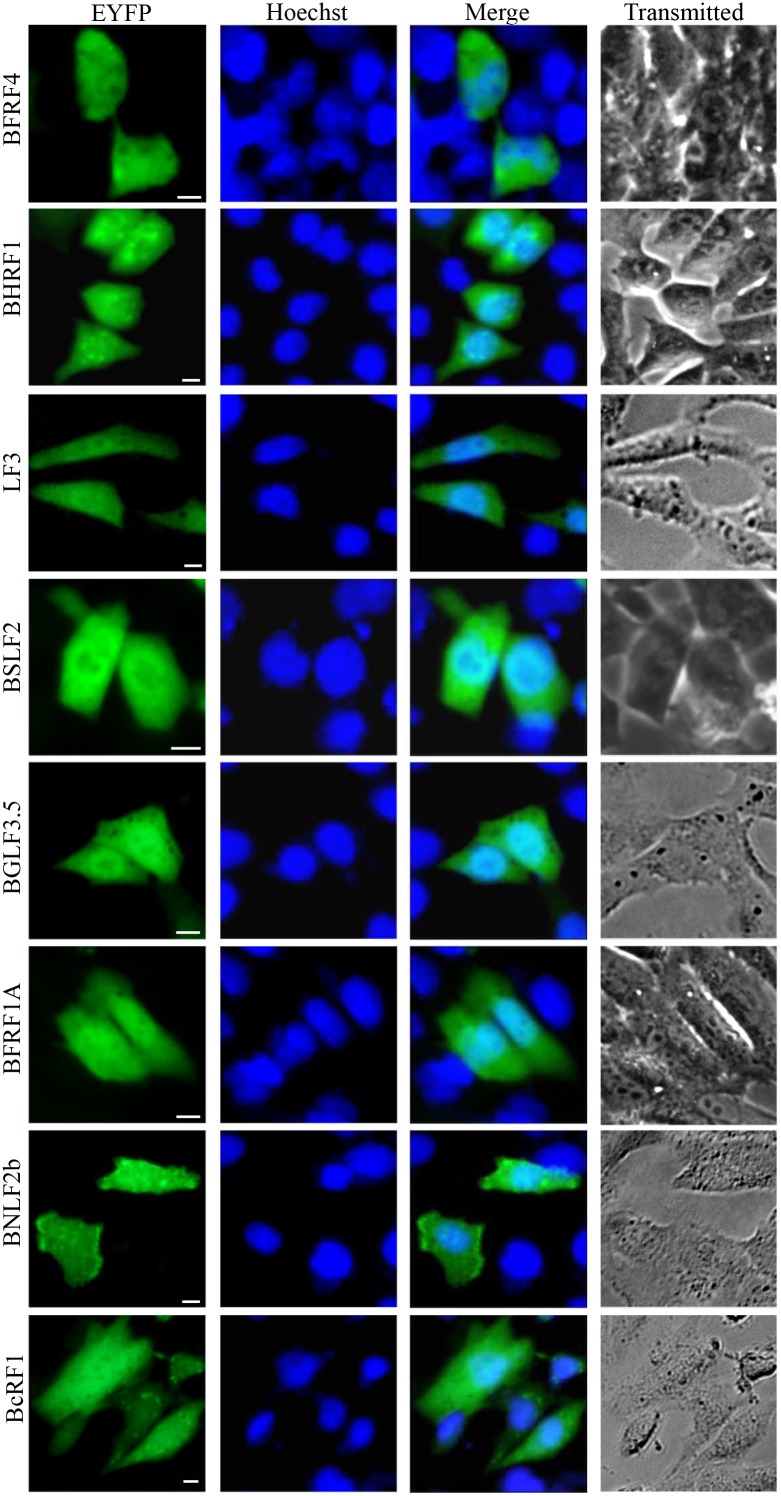

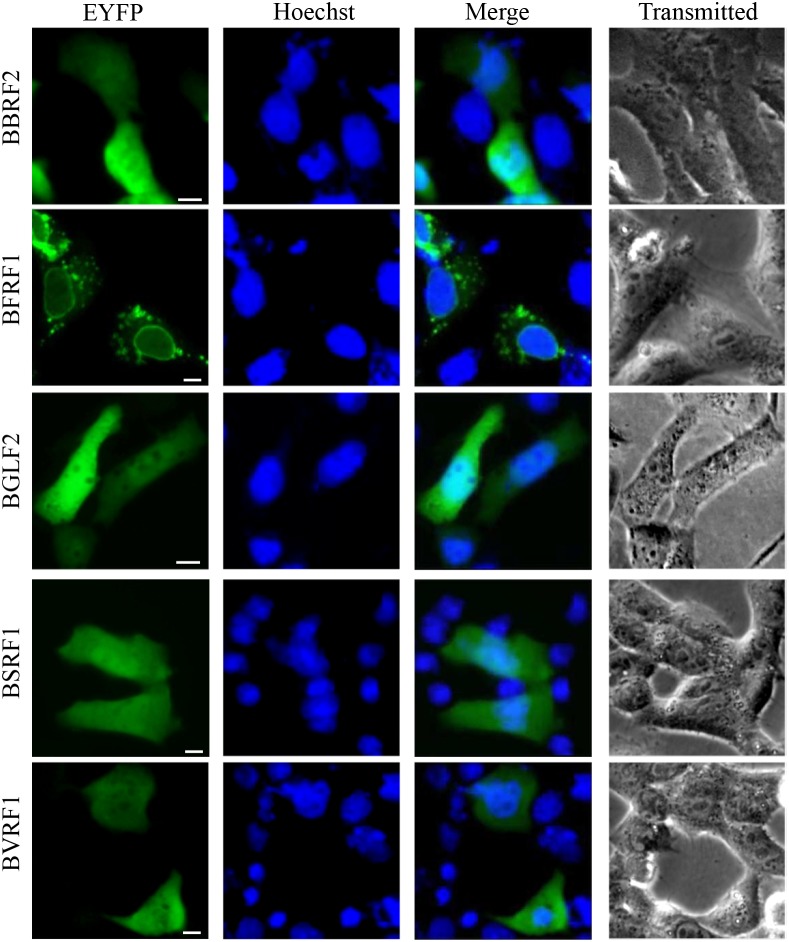
Pan-cellular localization summary of EBV-encoded proteins 21 EYFP-fused EBV proteins were expressed in COS-7 cells, and cells were subjected to fluorescence microscope analysis in live cells 24 h after transfection. Pictures were obtained using a Zeiss Axiovert 200M microscope. The same magnification was used in all panels. Representative fluorescence images of the vast majority live cells expressing indicated fusion protein were shown. Cells were counterstained with Hoechst to visualize the nuclear DNA. All scale bars indicate 10 μm.

**Figure 4:**
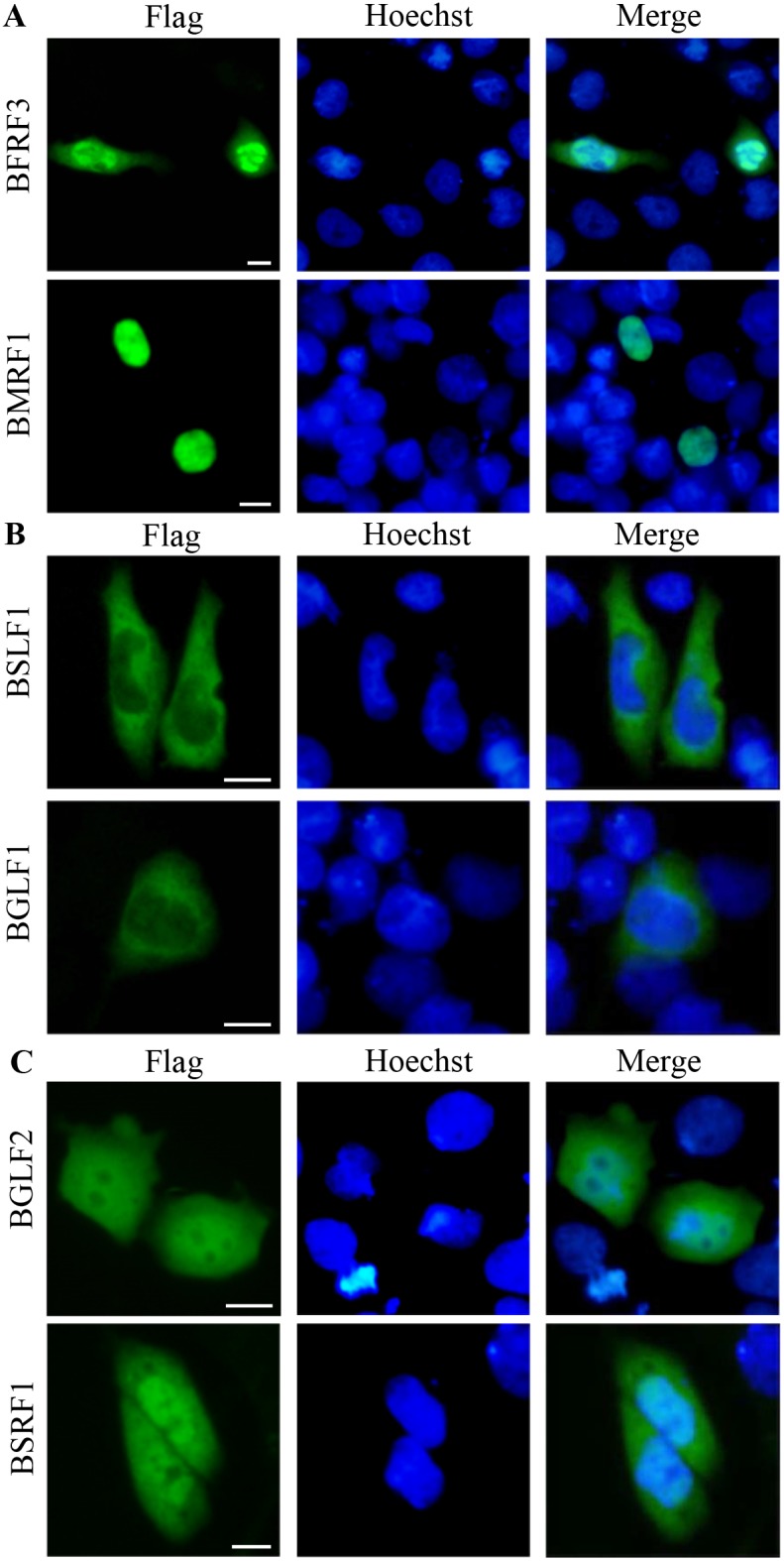
Verification the subcellular localization of some EBV representative proteins fused with Flag tag from each category (Tables 1 to 3) using IFA The plasmids expressing Flag fused BFRF3 and BMRF1 from nuclear localization, BSLF1 and BGLF1 from cytoplasmic localization, BGLF2 and BSRF1 from pan-cellular localization were transfected into COS-7 cells, and cells were subjected to IFA 24 h after transfection using anti-Flag mAb and FITC-conjugated goat anti-mouse IgG. Pictures were obtained using a Zeiss Axiovert 200M microscope. The same magnification was used in all panels. Representative fluorescence images of the vast majority live cells expressing indicated fusion protein were shown. Cells were counterstained with Hoechst to visualize the nuclear DNA. All scale bars indicate 10 μm.

Protein localizations were broadly classified as pan-cellular (means protein diffusely localized through-out the cytoplasm and nucleoplasm), pan-cytoplasmic (means protein diffusely localized through-out the cytoplasm) or subcytoplasmic (means protein can form spots or concentrated at some subcellular organelles in the cytoplasm), pan-nuclear (means protein diffusely localized through-out the nucleoplasm) or subnuclear (means protein can form spots or concentrated at some subcellular organelles in the nucleus). Some proteins possess more complicated localization patterns and can be divided into multiple categories and, in these cases, the protein was presented in the most prevailing category, since they certainly have the capability to form subcellular structures in some instances, perhaps at higher expression levels. A comprehensive list of all proteins expressed in this study are summarized in Figures 1 to 4 and detailed in Tables 1 to 3. In terms of their localizations, the EBV-encoded proteins could be generally fell into three groups: 28 proteins (34.57%) with nuclear or subnuclear localization (Figure 1 and Table 1), 32 proteins (39.51%) with cytoplasmic or subcytoplasmic localization (pan-cytoplasmic) (Figure 2 and Table 2), and 21 proteins (25.93%) were localized in both the cytoplasm and the nucleus (pan-cellular) (Figure 3 and Table 3). As a control, the fluorescence of EYFP from cells transfected with pEYFP-C1 was presented evenly distributed throughout the cytoplasm and the nucleoplasm but not the nucleolar structures (Figure 1).

**Table 1:** Nuclear localization summary of EBV-encoded proteins

No.	Protein name(s)	Function/description ^#^	COS-7 subcellular localization	Literature localization and references
1	BORF1 ^c^	Assembly/maturation, minor capsid protein-binding protein (mCP-BP), homology with triplex protein HSV-1 VP19C, VZV ORF20, MCMV M46 and KSHV ORF62	Subnuclear	Pan-nuclear ^***a***^ [6] Speckles in the nucleus ^***b***^ [48]
2	BSLF2/BMLF1 ^t^ (EB2, Mta, SM)	mRNA-export factor, mRNA splicing, interaction with human Spen proteins, homology with HSV-1 UL54 (ICP27), VZV ORF4, MCMV M69 and KSHV ORF57	Subnuclear, nucleolus like	N/A
3	EBNA3A ^l^ (BLRF3/BERF1)	Latency nuclear antigen 3A, negative effect on transactivator EBNA-2 and cell cycle, essential for immortalization	Pan-nuclear, without nucleolus	Pan-nuclear ^***a***^ [6, 49]
4	BZLF1 ^t^ (Zta, Z, Zebra, EB1)	Trans-activator ZEBRA, origin binding protein (EB1, Zta), bZip similar to CCAAT/enhancer binding protein, homology with KSHV K08	Pan-nuclear, without nucleolus	Pan-nuclear ^***a***^ [6] Nuclear ^***b***^ [50]
5	RPMS1 ^u^	Unknown function	Subnuclear, nucleolus like	Pan-nuclear ^***a***^ [34, 51]
6	BMLF1 ^t^ (Mta)	Immediate-early transactivator, part of BSLF2/BMLF1 protein	Subnuclear, nucleolus like	Nuclear and speckles in the nucleus ^***a***^ [52, 53] Nuclear ^***b***^ [50]
7	BXRF1 ^u^	Nucleoprotein, homology with HSV-1 UL24, VZV ORF35, MCMV M76 and KSHV ORF20	Subnuclear, nucleolus like	N/A
8	BALF2 ^r^	Major ssDNA binding protein, tegument protein, part of replication fork / machinery, homology with HSV-1 UL29, VZV ORF29, MCMV M57 and KSHV ORF6	Pan-nuclear, without nucleolus	Pan-nuclear ^***a***^ [6]
9	BMRF1 ^r^	DNA polymerase processivity factor, transcriptional transactivators, sliding clamp, early antigen protein D (EA-D, polymerase accessory protein), homology with HSV-1 UL42, VZV ORF16, MCMV M44 and KSHV ORF59	Pan-nuclear	Pan-nuclear ^***a***^ [6]
10	BLLF2 ^T^	Hypothetical protein, unknown function	Subnuclear, nucleolus like	Pan-nuclear ^***a***^ [6]
11	BFLF2 ^m^	Nuclear membrane phosphoprotein, part of intracellular virions, egress protein, herpesvirus UL31-like protein, complex with BFRF1, homology with HSV-1 UL31, VZV ORF27, MCMV M53 and KSHV ORF69	Pan-Nuclear	Nuclear ^***a***^ [54]
12	BdRF1 ^c^	Capsid scaffolding protein, homology with HSV-1 UL26.5, VZV ORF33.5, MCMV M80.5 and KSHV ORF17.5	Pan-nuclear, without nucleolus	N/A
13	EBNA1 ^l^ (BKRF1)	Latency nuclear antigen 1, assures EBV episome maintenance replication, Gly-rich domain, essential for immortalization	Pan-nuclear	Nuclear ^a/b^ [55]
14	BLLF3 ^n^	dUTP pyrophosphatase, dUTPase, homology with HSV-1 UL50, VZV ORF8, MCMV M72 and KSHV ORF54	Nuclear obviously > Cytoplasmic, without nucleolus	Pan-Nuclear ^a^ [6]
15	BGLF5 ^r^	Alkaline exonuclease, involved together with BALF2 in DNA recombination, homology with HSV-1 UL12, VZV ORF48, MCMV M98 and KSHV ORF37	Nuclear obviously > Cytoplasmic, without nucleolus	Nucleus and cytoplasm ^a^ [56] Nuclear ^a^ [57]
16	BRRF1 ^t^	Na, transcription factor, enhancement of the induction of the lytic cycle, homology with KSHV ORF49	Nuclear obviously > Cytoplasmic, without nucleolus	Nuclear ^a^ [58]
17	BCRF2 ^u^	Unknown function	Nuclear obviously > Cytoplasmic, without nucleolus	N/A
18	BDLF4 ^u^	Putative metal binding protein (gp115), homology with MCMV M92 and KSHV ORF31	Nuclear > Cytoplasmic	Pan-Cellular ^a^ [6]
19	BARF0 ^u^	Start at amino acid 298 of SWISS-PROT, homology with rhesus LCV	Nuclear obviously > Cytoplasmic	Nuclear ^a^ [59, 60]
20	BBLF1 ^T^	Myristoylated phosphoprotein in tegument (MyrP), homology with HSV-1 UL11, VZV ORF49, MCMV M99 and KSHV ORF38	Nuclear obviously > Cytoplasmic, without nucleolus	Pan-Cellular or Cytoplasmic > Nuclear ^a^ [6] trans-Golgi network (TGN) ^a/b^ [20]
21	BFRF3 ^c^	Smallest capsid protein (sCP) on outer capsid surface, homology with HSV-1 UL35 (VP26), VZV ORF23, MCMV M48.2 and KSHV ORF65	Nuclear obviously > Cytoplasmic, without nucleolus	Pan-Nuclear ^a^ [6] Nuclear ^b^ [61]
22	BGLF3 ^T^	gp118, not included in virions, homology with HSV-1 UL14, VZV ORF46, MCMV M95 and KSHV ORF34	Nuclear obviously > Cytoplasmic	Nuclear > Cytoplasmic ^a^ [6]
23	BGLF4 ^n^	Ser/Thr kinase, phosphorylation of nucleoside analogues, homology with HSV-1 UL13, VZV ORF47, MCMV M97 and KSHV ORF36	Nuclear obviously > Cytoplasmic	Nuclear ^a^ [62] Nuclear > Cytoplasmic ^a^ [6] Nuclear, a small portion is distributed in the cytoplasm at the late stage of virus replication ^b^ [63]
24	BKRF4 ^T^	Tegument phosphoprotein, homology with KSHV ORF45	Nuclear obviously > Cytoplasmic some with multiple small foci in the nucleus	Pan-Nuclear ^a^ [6]
25	BRLF1 ^t^ (Rta, R)	Transcriptional activator dimeric (TAF50), homology with KSHV ORF50	Nuclear obviously > Cytoplasmic, Cytoplasmic > Nuclear, Pan-cellular	Pan-Nuclear, Pan-Cytoplasmic, Pan-Cellular ^a^ [6, 64] Cytoplasmic and nuclear ^b^ [50]
26	BLRF2 ^T^	Tegument protein, homology with KSHV ORF52	Nuclear > Cytoplasmic, with speckles	Nuclear > Cytoplasmic ^a^ [6] Relocalized from the nucleus to the cytoplasm ^b^ [19]
27	BVRF2 ^c^	Proteinase/scaffold protein, capsid maturational protease, homology with HSV-1 UL26, VZV ORF33, MCMV M80 and KSHV ORF17	Nuclear slightly > Cytoplasmic	Pan-Nuclear ^a^ [6]
28	BOLF1 ^T^	Tegument large tegument protein-binding protein (LTPBP)	Nuclear obviously > Cytoplasmic, without nucleolus	N/A

a: transfection

b: infection

N/A: Not Detected

r: replication related protein

n: nucleotide metabolism related protein

t: transcription related protein

p: packaging related protein

l: latency related protein

m: membrane (or glycosylated) protein

c: capsid related protein

T: tegument protein

u: unkown function protein

^#^ Function/Description column compiled the following references: Tarbouriech et al. [5] and Salsman et al. [6].

**Table 2:** Cytoplasmic localization summary of EBV-encoded proteins

No.	Protein name(s)	Function/description ^#^	COS-7 subcellular localization	Literature localization and references
1	BILF2 ^m^	Potential membrane glycoprotein gp55/80, Ig-like, gp78	Subcytoplasmic, in intense perinuclear, ER or Golgi like	N/A
2	BLRF1 ^m^	Membrane glycoprotein gN, part of the gM-gN complex, part of the envelope-tegument interaction, homology with HSV-1 UL49A, VZV ORF9a, HHV5 UL73, MCMV M73 and KSHV ORF53	Subcytoplasmic, ER like	Subcytoplasmic, ER-like ^***a***^ [6]
3	BXLF2 ^m^	Glycoprotein H, gp85, part of gHgLgp42, homology with HSV-1 UL22, VZV ORF37, MCMV M75 and KSHV ORF22	Pan-Cytoplasmic	Cytoplasm and nuclear rim ^***a***^ [65]
4	BLLF1 ^m^	Envelope glycoprotein gp340/gp220, gp350, initial cell binding through complement receptor 2 (CR2, CD21)	Subcytoplasmic, perinuclear concentration, ER or Golgi like,	Golgi and plasma membrane ^***b***^ [20, 66]
5	BKRF2 ^m^	Glycoprotein L, gp25, in gL-gH complex involved in viral fusion together with gB, homology with HSV-1 UL1, VZV ORF60, MCMV M115 and KSHV ORF47	Subcytoplasmic, in intense perinuclear	N/A
6	BFLF1 ^p^	Major envelope protein, plays role in DNA packaging, cytosolic zinc-binding protein, cysteine rich, homology with HSV-1 UL32, VZV ORF26, MCMV M52 and KSHV ORF68	Pan-Cytoplasmic	N/A
7	BBLF2/3 ^r^	Primase-associated factor, spliced, full sequence not in DB, part of helicase–primase complex	Pan-Cytoplasmic	Pan-Cellular, Cytoplasmic > Nuclear ^***a***^ [6] Cytoplasmic localization or mixed cytoplasmic plus nuclear localization ^***a***^ [67]
8	BNLF1 ^l^ (LMP1)	Latent membrane protein, interferes with signalling, TRAF-binding through CTAR1 and 2, essential for immortalization	Subcytoplasmic, with speckle structures	Cytosolic, cytoskeletal, lipid raft and internal perinuclear membranes ^***a***^ [68]
9	LMP2A ^l^	Latent membrane protein, interference with protein kinase signalling, gene terminal protein, essential for immortalization, homology with KSHV K15	Subcytoplasmic, perinuclear speckle concentration	Perinuclear regions, plasma membrane ^***a***^ [69]
10	LMP2B ^l^	Latent membrane protein, negative regulator of LMP-2A	Subcytoplasmic, nuclear membrane, like trans-Golgi network	Perinuclear regions and trans-Golgi network ^***a***^ [69, 70]
11	BCRF1 ^t^ (vIL-10)	Viral interleukin-10 homologue precursor, vIL-10, homology with MCMV M87 and KSHV ORF24	Pan-Cytoplasmic	Subcytoplasmic ^***a***^ [6]
12	BRRF2 ^T^	Unknown function, homology with KSHV ORF48	Pan-Cytoplasmic	Subcytoplasmic ^***a***^ [6]
13	BSLF1 ^n^	Primase, DNA helicase-primase component, homology with HSV-1 UL52, VZV ORF6, MCMV M70 and KSHV ORF56	Pan-Cytoplasmic	Pan-cytoplasmic ^***a***^ [6]
14	BFRF2 ^u^	Possible capsid protein, not included in virions, unknown function, homology with MCMV M49 and KSHV ORF66	Pan-Cytoplasmic	Pan-cytoplasmic ^***a***^ [6]
15	BBRF1 ^c^	Minor capsid protein portal protein UL6 homologue, homology with HSV-1 UL6, VZV ORF54, MCMV M104 and KSHV ORF43	Subcytoplasmic, with speckles in the cytoplasmic	Pan-cytoplasmic ^***a***^ [6]
16	BMRF2 ^m^	Receptor for cellular integrins, needed for infection of epithelial cells, 10 TM helices, RGD motif, membrane accociated protein, homology with KSHV ORF58	Subcytoplasmic, cytoplasmic obviously > Nuclear, perinuclear concentration, ER or Golgi like	Cytoplasm and membrane ^a/b^ [71] ER and Golgi ^a^ [72]
17	BALF1 ^t^	Bcl-2 homologue, negative regulator of anti-apoptosis protein BHRF1	Pan-Cytoplasmic	Cytoplasmic > Nuclear ^a^ [6] Cytoplasmic ^a^ [73]
18	ECRF4 ^u^	Unkown function	Cytoplasmic obviously > Nuclear	N/A
19	BALF4 ^m^	Glycoprotein B, glycoprotein 110, fusion and co-receptor binding, homology with HSV-1 UL27 (gB), VZV ORF31, MCMV M55 and KSHV ORF8	Cytoplasmic obviously > Nuclear, intense in perinuclear cytoplasmic with some patches, ER or Golgi like	Cytoplasmic, perinuclear and nuclear membranes ^a^ [74] Cytoplasmic, nuclear membranes, ER and the inner/outer nuclear membranes, plasma membrane and cell surface ^b^ [66, 75]
20	BILF1 ^m^	G-protein coupled receptor (G-PCR), includes 7 transmembrane helices, 6 glycosylation sites, 2 disulfide bridges gP64	Pan-Cytoplasmic, plasma membrane like	Plasma membrane ^a^ [76, 77] Cytoplasmic membrane ^b^ [78]
21	BARF1 ^t^	CD80 homologue (p33), oncogene, soluble glycoprotein, includes 2 Ig domains, binds CSF-1, homology with HSV-1 UL40, VZV ORF18 and KSHV ORF60	Cytoplasmic obviously > Nuclear	Cytoplasmic membrane ^a/b^ [79] Subcytoplasmic, ER-like, perinuclear concentration ^a^ [6]
22	BVLF1 ^u^	Unknown function	Cytoplasmic obviously > Nuclear	N/A
23	LF1 ^u^	Contains a dUTPase like domain, γ-herpes ORF10 family	Cytoplasmic obviously > Nuclear	N/A
24	BNLF2a ^u^	Potential membrane protein	Cytoplasmic obviously > Nuclear, ER or Golgi like	Pan-Cytoplasmic ^a^ [6] ER ^a^ [80]
25	BGLF1 ^T^	Potential tegument protein (gp115), homology with HSV-1 UL17, VZV ORF43, MCMV M93 and KSHV ORF32	Pan-Cytoplasmic	Pan-Cytoplasmic ^a^ [6]
26	BORF2 ^n^	Ribonucleotide-reductase, large subunit, 140 kDa, homology with HSV-1 UL39, VZV ORF19, MCMV M45 and KSHV ORF61	Cytoplasmic obviously > Nuclear, with punctates in the cytoplasmic	Subcytoplasmic, punctate cytoplasmic crystal-like structures ^a^ [6]
27	BXLF1 ^n^	Thymidine kinase, homology with HSV-1 UL23, VZV ORF36 and KSHV ORF21	Subcytoplasmic, irregularly shaped cytoplasmic structures	Subcytoplasmic, irregularly shaped cytoplasmic structures ^a^ [6]
28	BBLF4 ^r^	Helicase, part of helicase–primase complex, homology with HSV-1 UL5, VZV ORF55, MCMV M105 and KSHV ORF44	Cytoplasmic obviously > Nuclear	Cytoplasmic > Nuclear ^a^ [81]
29	BALF5 ^r^	DNA polymerase, homology with HSV-1 UL30, VZV ORF28, MCMV M54 and KSHV ORF9	Pan-Cytoplasmic	Nuclear ^b^ [21]
30	BTRF1 ^c^	Hypothetical protein, involved in capsid maturation, capsid-associated, homology with HSV-1 UL21, VZV ORF38, MCMV M88 and KSHV ORF23	Pan-Cytoplasmic	Nuclear > Cytoplasmic ^a^ [6]
31	LF2 ^u^	Contains a dUTPase like domain, γ-herpes ORF11 family	Cytoplasmic, cytoskeletal like	Cytoplasmic, cytoskeletal ^***a***^ [82]
32	BDLF2 ^T^	Potential type II glycosylated envelope protein, homology with KSHV ORF27	Subcytoplasmic, with perinuclear concentration	Subcytoplasmic, ER-like ^***a***^ [6, 72]

a: transfection

b: infection

N/A: Not Detected

r: replication related protein

n: nucleotide metabolism related protein

t: transcription related protein

p: packaging related protein

l: latency related protein

m: membrane (or glycosylated) protein

c: capsid related protein

T: tegument protein

u: unkown function protein

^#^ Function/Description column compiled the following references: Tarbouriech et al. [5] and Salsman et al. [6].

**Table 3:** Pan-cellular localization summary of EBV-encoded proteins

No.	Protein name(s)	Function/description ^#^	COS-7 subcellular localization	Literature localization and references
1	BDLF1 ^c^	Minor capsid protein, homology with HSV-1 UL18 (VP23), VZV ORF41, MCMV M85 and KSHV ORF26	Pan-Cellular, without nucleolus	Pan-Cellular ^***a***^ [6, 48]
2	BBRF3 ^m^	Integral membrane protein glycoprotein M, part of gN-gM complex involved in envelope-tegument interaction, homology with HSV-1 UL10, VZV ORF50, MCMV M100 and KSHV ORF39	Pan-Cellular	N/A
3	BDLF3 ^m^	Membrane glycoprotein (gp150), gp117, homology with KSHV ORF28	Pan-cellular, with punctate perinuclear and cytoplasmic	Subcytoplasmic, punctate perinuclear concentration ^***a***^ [6]. Cytoplasmic ^***b***^ [83]
4	BZLF2 ^m^	gp42, MHC class II binding protein, part of gHgLgp42 complex	Plasma membrane	N/A
5	BGRF1/BDRF1 ^p^	DNA-packaging protein, terminase small subunit, homology with HSV-1 UL15, VZV ORF42, MCMV M89 and KSHV ORF29a	Pan-Cellular	N/A
6	BWRF1 ^l^	EBNA-Lp (EBNA-5) nuclear phosphoprotein, highly spliced, 12 exons, proline-rich, enhances EBNA-2 transactivation	Pan-Cellular, with speckles in cytoplasm and perinuclear	N/A
7	BaRF1 ^n^	Ribonucleotide reductase, small 38 kDa subunit, homology with HSV-1 UL40, VZV ORF18 and KSHV ORF60	Pan-Cellular	Pan-Cellular ^***a***^ [6]
8	BKRF3 ^n^	Uracil DNA glycosylase, homology with HSV-1 UL2, VZV ORF59, MCMV M114 and KSHV ORF46	Pan-Cellular	Pan-Cellular ^***a***^ [6, 84] Translocated from the cytoplasm into the nucleus ^***b***^ [21]
9	BFRF4 ^p^	DNA-cleavage and packaging protein, part of the DNA packaging machinery (BFRF0.5, HS4BAM), homology with HSV-1 UL33, VZV ORF25, MCMV M51 and KSHV ORF67.5	Pan-Cellular	N/A
10	BHRF1 ^t^	Early antigen protein R (EA-R), nuclear antigen, anti-apoptotic factor Bcl-2 homologue	Pan-Cellular, with speckles in cytoplasm and perinuclear, mitochondria like	Mitochondria, ER and nuclear membranes ^***a***^ [85] Mitochondria ^***b***^ [86]
11	LF3 ^u^	Unknown function	Pan-Cellular	N/A
12	BSLF2 ^u^	N-terminal fragment (5' exon) of SM/EB2 protein and part of BSLF2/BMLF1 protein, homology with HSV-1 UL54, VZV ORF4, MCMV M69 and KSHV ORF57	Pan-Cellular, without nucleolus	Pan-Cellular ^***a***^ [6, 48]
13	BGLF3.5 ^u^	Unknown function, homology with MCMV M96 and KSHV ORF35	Pan-Cellular, without nucleolus	N/A
14	BFRF1A ^u^	Unknown function	Pan-Cellular, without nucleolus	N/A
15	BNLF2b ^u^	Potential gp141	Pan-Cellular, cell membrane	N/A
16	BcRF1 ^u^ (UL87)	Unknown function, herpesvirus UL87 family, homology with MCMV M87 and KSHV ORF24	Pan-cellular, with speckle cytoplasmic structures	Subcytoplasmic ^***a***^ [6]
17	BBRF2 ^T^	Unknown function, homology with HSV-1 UL7, VZV ORF53, MCMV M103 and KSHV ORF42	Pan-Cellular	Pan-Cellular ^***a***^ [6]
18	BFRF1 ^m^	Nuclear membrane protein p38, transmembrane with large cytoplasm domain, complex with BFLF2, homology with HSV-1 UL34, VZV ORF24, MCMV M50 and KSHV ORF67	Subcytoplasmic and perinuclear region, with speckle cytoplasmic structures	Pan-Cytoplasmic and perinuclear region ^***a***^ [6, 87] Nuclear envelope ^***b***^ [88]
19	BGLF2 ^T^	MyrPBP, homology with HSV-1 UL16, VZV ORF44, MCMV M94 and KSHV ORF33	Pan-Cellular, without nucleolus	Pan-Cellular ^***a***^ [6] Nuclei with granular ^***b***^ [89]
20	BSRF1 ^T^	Palmitoylated tegument protein (PalmP), homology with HSV-1 UL51, VZV ORF7, MCMV M71 and KSHV ORF55	Pan-Cellular	Subcytoplasmic, perinuclear concentration ^***a***^ [6]
21	BVRF1 ^T^	Capsid associated protein, portal plug (EC-RF2), seals DNA inside capsid, homology with HSV-1 UL25, VZV ORF34, MCMV M77 and KSHV ORF19	Pan-Cellular	Pan-Cellular, Cytoplasmic > Nuclear ^***a***^ [6]

a: transfection

b: infection

N/A: Not Detected

r: replication related protein

n: nucleotide metabolism related protein

t: transcription related protein

p: packaging related protein

l: latency related protein

m: membrane (or glycosylated) protein

c: capsid related protein

T: tegument protein

u: unkown function protein

^#^ Function/Description column compiled the following references: Tarbouriech et al. [5] and Salsman et al. [6].

Of the 81 viral proteins we tested, 20 EBV proteins have not been previously characterized with respect to their localization and most of these proteins also lack any functional identification, 52 EBV proteins have previously published subcellular localization data, and these results are in consonance with previous reports (see Tables 1 to 3 for individual protein results). Meanwhile, minor discrepancies in localization were detected for 4 proteins (BLLF2, BKRF4, BLRF2 and BBRF1) diverse from previous studies, however, significant discrepancies in localization were observed for 5 proteins (BDLF4, BSRF1, BBLF1, BALF5 and BTRF1) distinct from previous results. Moreover, the subcellular localizations of some Flag-fused representative proteins from each category (BFRF3 and BMRF1 from nuclear localization, BSLF1 and BGLF1 from cytoplasmic localization, BGLF2 and BSRF1 from pan-cellular localization) were consistent with the subcellular localizations of those fused with EYFP tag (Figure 4), further making the results more convincing.

Some discrepancies could not be unexpected with localizations assessed in the course of viral infection since the presence of interplays between viral proteins can change the localization of the individual proteins. The localization discrepancies of specific proteins might also be associated with protein expression levels or the presence of a tag. Not surprised, this initial analysis demonstrated that such high through-put localization screening will not precisely clarify the localization of every viral protein in the context of infection, but it did present that such an approach is suitable for analyzing the localization of the vast majority of viral proteins even during viral infection.

## DISCUSSION

Herpesviruses are large DNA viruses that encode a variety of proteins for complicated interactions with host. For the sake of taking an investigation on the possible roles of the many uncharacterized EBV-encoded proteins, the genomic expression libraries of EBV were constructed and screened to explore the complete intracellular localization map of almost all EBV proteins in mammalian cells. However, when we were cloning EBV genes, the subcellular localizations of 61 from 81 proteins have been probed by others to our knowledge (Tables 1 to 3), and with only slight differences, our results were good correlated with previously reported protein localizations, while new localization data was yielded for approximately 20 previously unlocalized proteins.

Almost one-third of EBV-encoded proteins localized principally in the nucleus (28 proteins) (Figure 1 and Table 1). Furthermore, 32 proteins primarily showed cytoplasmic or subcytoplasmic localization (Figure 2 and Table 2), and others localized throughout both compartments (often at unequal levels, 21 proteins) (Figure 3 and Table 3). It is of interest that 34.57% of the EBV-encoded proteins were detected in the nucleus, whereas only 12% of randomly selected cellular proteins showed nuclear localization [17]. A recent genome-wide subcellular localization study reported that human herpesvirus 8 (HHV-8, gamma-herpesvirus) was found to have 51% cytoplasmic and 22% nuclear proteins (with 27% in both compartments) [18], indicates that EBV has a higher proportion of nuclear proteins than HHV-8. Nuclear predominance of EBV-encoded proteins is in good consonance with the viral life cycle, which is preferentially associated with the nucleus.

In the present study, some of the results from transfection may be different from infection, because of the interactions of viral proteins during infection (data as shown in Tables 1 to 3). While these transfection results might not uncover the accurate subcellular localization of every viral proteins during infection, the subcellular localization map of individually expressed EBV-encoded proteins could offer helpful data to further examine the mechanism by which individual EBV-encoded protein effects on EBV pathogenesis.

Minor discrepancies were observed for only 4 proteins (BLLF2, BKRF4, BLRF2 and BBRF1) in this study [6, 19]. Specifically, BLLF2 and BKRF4 were detected exclusively in the nucleus by other investigators, whereas in our study BLLF2 showed subnuclear localization (nucleolus like), and BKRF4 showed obviously nuclear localization with multiple small foci. The minor capsid protein BBRF1 showed subcytoplasmic (with speckles in the cytoplasmic), which is different from the pan-cytoplasmic localization in previous reports. BLRF2 showed the fluorescence of nucleus is more than cytoplasm (with speckles), but this contrary to previous study in plasmid transfection, which may relocalized from the nucleus to the cytoplasm relied on the interaction with other viral proteins in the course of viral infection.

Significant differences were also observed for 5 proteins (BDLF4, BSRF1, BBLF1, BALF5 and BTRF1). In our study, BDLF4 showed the fluorescence in nucleus is more than cytoplasm, which is different from the pan-cellular localization reported by others [6]. The myristoylated phosphoprotein BBLF1 was found obviously nuclear localization without nucleolus that different with pan-cellular or cytoplasmic localization in previous study, which may depend on its myristoylation modification [6, 20]. BSRF1 showed pan-cellular localization, whereas it gave a subcytoplasmic or perinuclear concentration localization in a previous report [6], which may be explained by subtle difference in employ of a different cell lines. The DNA polymerase BALF5 localized exclusively in the cytoplasm, which is different from the predominantly nuclear localization during EBV infection [21]. It’s reported that the nuclear transport of HHV-8 DNA polymerase holoenzyme is dependent on the nuclear localization signal (NLS) present on the processivity factor PF-8, since the catalytic subunit pol-8 lacks a functional NLS, and hence the two subunits are targeted into the nucleus as a complex [22]. Therefore we speculated that the transport of BALF5 from cytoplasm to nucleus requires the expression of additional viral factors, which could help BALF5 target into the nucleus for executing its function in viral DNA synthesis. In addition, BTRF1 was also detected only in the cytoplasm, which is demonstrated to be a nuclear-targeted protein (nuclear > cytoplasmic) previously.

Latency is a regulatory status, which may mainly rely on nuclear proteins to manipulate host cell and viral transcription. Compared with the intracellular localization map of HHV-8 [18], which found that all latency-associated proteins showed a nuclear staining pattern, we detected only two latent related proteins EBNA3A (BLRF3/BERF1) and EBNA1 (BKRF1) localized in the nucleus, whereas the nuclear phosphoprotein BWRF1, latent membrane protein BNLF1 (LMP1), LMP2A and LMP2B showed pan-cellular (with speckles in cytoplasm and perinuclear), subcytoplasmic (with speckle cytoplasmic structures), perinuclear speckle concentration and nuclear membrane (like trans-Golgi network) localization, respectively. This disparity may be due to the different viral life cycle between EBV and HHV-8.

The assembly compartment for viral proteins in the host cell is probably relevant to its subcellular localization. EBV contains 13 membrane related protein, and 7 glycoproteins (BLLF1/gp350, BXLF2/gH, BALF4/gB, BBRF3/gM, BLRF1/gN, BDLF3/gp150 and BKRF2/gL) are incorporated into its envelope [5, 8], which play crucial roles during virus infection. Besides, BILF2/gp55/80, a predicted membrane protein, also perhaps targets to the envelope. In this study, most of these proteins exhibited pan-cytoplasmic, subcytoplasmic, membraneous or pan-cellular localization, without nuclear or subnuclear localization (Figures 2 and 3, Tables 2 and 3). This is high concordance with the compartment where the envelope assembling occurs. Other 4 membrane proteins (BZLF2/gp42, BILF1/G-PCR, BMRF2 and BFRF1) also showed plasma membrane, pan-cytoplasmic or subcytoplasmic and perinuclear localization (with speckle cytoplasmic structures), but only BFLF2 localized absolutely to the nucleus, which is in accordance with the subcellular localization of its homologue HSV-1 UL31.

In addition, it has been established that EBV virion has 6 proteins in its capsid (BDLF1, BORF1, BBRF1, BVRF2, BdRF1 and BFRF3) [5, 8], and BTRF1 is also predicted to be a potential protein that implicated in capsid maturation. In this work, BVRF2 and BFRF3 displayed enriched localization in the nucleus, and BORF1 (subnuclear) and BdRF1 showed complete localization in the nucleus, where the capsid assembling takes place. However, BDLF1, BBRF1 and BTRF1 demonstrated pan-cellular, pan-cytoplasmic or subcytoplasmic localization.

It’s reported the replication of EBV involved in 6 proteins (BGLF5, BBLF2/3, BBLF4, BALF5, BALF2 and BMRF1) [5, 8]. Interestingly, BGLF5, BBLF4, BALF2 and BMRF1 showed slightly or obviously nuclear localization in our job, this is correlates with the fact that EBV DNA replication occurs in the nucleus. However, BBLF2/3 and BALF5 appeared pan-cytoplasmic localization, which may be transported into the nucleus for viral DNA replication under the interaction with other viral proteins during infection [21]. The viral nucleotide metabolism is also important for virus replication, which implicated in 7 proteins (BaRF1, BKRF3, BLLF3, BGLF4, BSLF1, BXLF1 and BORF2) [5, 8]. Here, BaRF1, BKRF3, BLLF3 and BGLF4 showed apparently nuclear localization or pan-cellular localization, whereas BSLF1, BXLF1 and BORF2 showed pan-cytoplasmic or subcytoplasmic localization.

After herpesviral DNA replication, the RNA transcription is taken place later in the nucleus, and the transcription of EBV is associated with 9 proteins (BHRF1, BALF1, BARF1, BCRF1, BRRF1, BSLF2/BMLF1, BZLF1, BRLF1 and BMLF1) [5, 8]. It is of interest that BHRF1, BARF1, BRRF1, BSLF2/BMLF1, BZLF1, BRLF1 and BMLF1 showed pan-cellular or clearly nuclear localization, whereas BALF1 and BCRF1 were absolutely distributed in the cytoplasm. Besides, all the packaging related protein (BFLF1, BFRF4 and BGRF1/BDRF1) showed pan-cytoplasmic or pan-cellular localization, which is consistent with the compartment where the viral packaging takes place.

EBV is the first known human tumor virus to play an essential role in the induction of a broad spectrum of human lymphoid and epithelial malignancies [23–26], yet the fundamental mechanism of how EBV contributes to cancer remain unknown. Unlike other herpesviruses, the development of EBV-related tumorous diseases is relevant with the latent cycle, by virtue of the immune system is incompetent to monitor latently infected cells. It’s shown that EBV can encode some viral oncoproteins that involved in EBV tumorigenicity (including EBNA1, EBNA2, LMP1, LMP2, EBNA3A, EBNA3C, BARF0, BALF1, RPMS1, BARF1 and BNLF1) [27–38], which are associated with Burkitt’s lymphoma (EBNA1, LMP2A and BARF0), Hodgkin’s disease, nasopharyngeal carcinoma and NK/T-cell lymphoma (EBNA1, LMP1, LMP2A and LMP2B), gastric carcinomas (BARF1), post-transplant lymphoproliferative disorders and AIDS-related lymphomas (EBNA1, EBNA3A, LMP1, LMP2A and LMP2B). It’s well known the subcellular localization plays a critical role in the function execution of a specific protein, and therefore we speculated the subcellular localization of these mentioned EBV proteins might take some potential roles in the EBV-related lymphoid and epithelial malignancies, e.g. the subcytoplasmic localization of LMP1 may be crucial for it to lead to changes that is connected with B-cell activation, including B-cell fusion, increase of CD23, CD39, CD40, CD44 expression and apoptosis-restraining effects [39, 40]. Furthermore, this localization may also important for LMP1 to promote oncogenesis and transformation of primary rodent fibroblasts and to impede differentiation of a squamous carcinoma cell line [41]. However, the exact pathological roles in EBV-related malignancies are not fully elucidated, this need further in-depth study.

In conclusion, this study on the construction of a library of expression clones for the EBV proteome, we believe, will be a remarkably essential work in producing highly valuable platform for further studies of the viral life cycle and mechanistic pathogenesis in the future. Additionally, it will also be applicable for screening the possible viral proteins or host cellular factors that may interact with viral proteins.

## MATERIALS AND METHODS

### Cell culture

COS-7 cells, incubated at 37°C in a humidified 5% CO2 incubator, were grown in Dulbecco’s modified MEM (DMEM, Gibco-BRL) supplemented with 10% fetal bovine serum (FBS; Gibco-BRL), 2 mM L-glutamine, and 50 U/ml penicillin G and 50 μg/ml streptomycin.

### Cloning of EBV genes

The enzymes used for cloning programs were purchased from Thermo Scientific except DNA polymerase KOD-Plus-Neo from TOYOBO and T4 DNA Ligase from Takara. The 81 ORFs of EBV from the NCBI entries, including the start methionine, were amplified by PCR from the BAC DNA of B95-8 strain of EBV (174-kb BAC) except LF1, LF2 and LF3 from the BAC DNA of Akata strain of EBV (AK-BAC) [4], using specific primers with suitable overhanging restriction enzyme motifs (contain *Hin*dIII and *Bam*HI sites unless otherwise specified). Due to EBV genome contains high GC, the annealing temperature for PCR reaction is generally high. EYFP has been widely employed as a reporter to visualize EYFP-tagged proteins in live cells. Therefore, the amplified DNA products were digested with *Hin*dIII and *Bam*HI and inserted into the multicloning site of pEYFP-C1 (Clontech, BD Biosciences) in frame with an EYFP tag at the C terminus, which is digested with appropriate restriction enzymes, with the aim to yield corresponding EYFP fusion protein to allow direct observation of the subcellular localization of each protein. Furthermore, some representative proteins from each category (BFRF3 and BMRF1 from nuclear localization, BSLF1 and BGLF1 from cytoplasmic localization and BGLF2 and BSRF1 from pan-cellular localization) were also subcloned into pCMV-Flag-N1 (Beyotime Biotechnology) in frame with a Flag tag at the N terminus. All constructs described above were verified by plasmid PCR, restriction analysis and full-length DNA sequencing, and all primers used in this research are available upon request.

### Plasmid transfection and fluorescence microscopy

To test the subcellular localization of EBV proteins in live cells, plasmid transfection and fluorescence microscopy assays were performed as described in our previous studies [42–46]. Briefly, COS-7 cells were plated onto 12 well plates (Corning, USA) and cultured in DMEM with 10% FBS overnight to reach the confluency 60-80% before transfection. The next day, monolayer cells were transfected with 1.5 μg of assigned plasmid DNA mixed with TurboFect Transfection Reagent (Thermo Scientific) as per the manufacturer’s instructions. After transfection for 24 h, the live cells were beard for fluorescence microscopy. To make the data more convincing, the subcellular localizations of some representative proteins fused with Flag tag from each category were detected by IFA, using anti-Flag monoclonal antibody (mAb) (ABmart) and fluorescein isothiocyanate (FITC)-conjugated goat anti-mouse IgG (Sigma-Aldrich), as described in previous studies [47]. In the same observation, each transfection was carried out for at least two times. Data shown were from one illustrative experiment. Fluorescences were analyzed using a Zeiss Axiovert 200 M inverted fluorescence microscope (Carl Zeiss, Germany), equipped with a halogen lamp (100 HAL, 12 V, 100 Watt) for transmitted light microscopy and an objective LD “Plan-Neofluar” with 40×/0.6 Corr M27 (D=0-1.5) lens (WD=3.3mm when D=0 and WD=2.5mm when D=1.5). The YFP (EX BP 500/20, BS FT 515, EM BP 535/30), FITC (EX BP 475/40, BS FT 500, EM BP 530/50) and DAPI (EX G 365, BS FT 395, EM BP 445/50) filtersets were used to detect EYFP labelled proteins, FITC labelled proteins and nuclear DNA labelled with Hoechst, respectively. Transmitted and fluorescence light images were captured under a digital camera (Axiocam; Carl Zeiss), with Zeiss AxioVision Rel. 4.8 software for controlling the image recording, microscope stage and image merge. Microscopic settings were kept constant for comparisons among different samples. All the pictures were taken under a magnification of 400×. Classification of subcellular localization of the proteins was determined by three researchers independently, and categorization was discussed until consensus was reached. Each picture represents most of the cells with similar subcellular localization. Light-translucent photomicrographs are introduced to show cellular morphology. Cells were counterstained with Hoechst to visualize the nuclear DNA. Fluorescent images of EYFP fusion proteins and FITC labelled protein were presented in pseudocolor green and genuine color green, respectively, and merged with Hoechst using Zeiss AxioVision Rel. 4.8 software. All scale bars indicate 10 μm, and images were processed using Adobe Photoshop.
